# Hepatitis B and Delta and the role of the Fundação de Medicina Tropical Dr. Heitor Vieira Dourado

**DOI:** 10.1590/0037-8682-0505-2025

**Published:** 2026-06-22

**Authors:** Arlene dos Santos Pinto, Yanka Karolinna Batista-Rodrigues, Ramon Peixoto de Castro, Bernardo Maia da Silva, Camila Helena Bôtto-Menezes, Marcia da Costa Castilho, Wornei Silva Miranda Braga

**Affiliations:** 1 Fundação de Medicina Tropical Doutor Heitor Vieira Dourado, Grupo de Pesquisa, Núcleo de Estudo das Viroses Humanas do Amazonas, Gerência de Virologia, Manaus, AM, Brasil.; 2 Universidade do Estado do Amazonas, Programa de Pós-graduação em Doenças Tropicais e Infecciosas, Manaus, AM, Brasil.; 3 Universidade Federal de Minas Gerais, Instituto de Ciências Biológicas, Departamento de Genética, Ecologia e Evolução, Laboratório de Biologia Integrativa, Grupo de Pesquisa em Bioestatística e Epidemiologia Molecular, Belo Horizonte, MG, Brasil.; 4 Universidade do Estado do Amazonas, Escola de Ciências da Saúde, Manaus, AM, Brasil.

**Keywords:** Hepatitis Delta Virus, Hepatitis B Virus, Coinfection, Liver diseases, Public Health Surveillance, Amazonia

## Abstract

Hepatitis B and D display a distinctive epidemiological pattern in the Western Amazon, disproportionately affecting Indigenous peoples and riverine populations across Brazil, Peru, Colombia, Venezuela, and Ecuador. Despite more than three decades of universal hepatitis B vaccination, moderate endemicity and a high burden of chronic carriers persist in the region. In the state of Amazonas, Brazil, the Fundação de Medicina Tropical Dr. Heitor Vieira Dourado (FMT-HVD) has played a pivotal historical and contemporary role in the recognition, diagnosis, clinical management, surveillance, and research of HBV and HDV infections, serving as a major reference center for the region. The local health-care network is aligned with national policies but faces substantial challenges related to the vast geographic territory, strong centralization of specialized services in Manaus, and limited availability of trained professionals in endemic and remote areas. Logistical, socioeconomic, and cultural barriers continue to restrict timely access to diagnosis and treatment, while universal vaccination, widespread rapid testing, and ongoing decentralization efforts represent key facilitating factors. Looking ahead, the proposed research agenda emphasizes integrated surveillance, genomic and clinical-epidemiological studies, evaluation of the care cascade, and strengthening of primary health care as essential strategies to reduce regional inequalities and accelerate progress toward the elimination of viral hepatitis by 2030.

## TEMPORAL TRENDS AND CURRENT STATUS OF HEPATITIS B AND DELTA IN THE STATE OF AMAZONAS

No text that seeks to describe the global impact of hepatitis B virus (HBV) and hepatitis D virus (HDV) can overlook the peculiar epidemiological patterns and idiosyncratic clinical features of these diseases among Indigenous peoples and riverine populations of the Western Amazon, spanning regions of Brazil^1^, Peru^2^, Venezuela^3^, Colombia^4^ and Ecuador^5^.

Even after more than thirty years of universal hepatitis B vaccination in these regions, moderate endemicity patterns persist^6^. Despite the clear impact of vaccination in reducing incidence rates and interrupting vertical HBV transmission - the primary mechanism reported^6^ - the number of chronic HBV and HDV carriers, as well as candidates for liver transplantation, continues to place a heavy burden on outpatient clinics in the smaller provinces and major capitals of northern Brazil.

Around 15 to 20% of hepatitis B cases in Brazil are reported annually from states in the northern region^7^. Mathematical modeling studies suggest that approximately 70% of cases in the country remain undiagnosed^8^. In our region, although notifications indicate significant occurrence in the state capitals, hepatitis B and Delta are also prevalent among riverine communities and Indigenous populations, which may negatively affect the detection of new cases and hinder the World Health Organization’s goal of controlling and eliminating viral hepatitis by 2030^9^.

**FIGURE 1:**
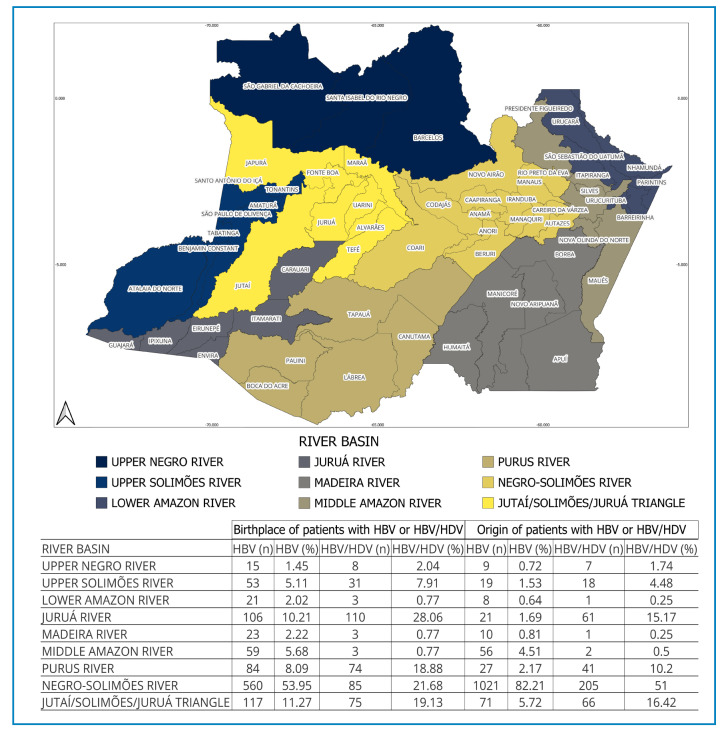
Geographical distribution of birthplace and place of actual living of patients with HBV and HBV/HDV attended at the Fundação de Medicina Tropical Dr. Heitor Vieira Dourado, Amazonas, Brazil (2012-2022). **Source:** Castro et al. Unpublished data NOTE: The figure is based on a retrospective dataset compiled as part of an ongoing study, including all patients with confirmed HBV or HBV/HDV infection who attended FMT-HVD during the study period. Data were obtained from clinical records and institutional laboratory databases. Percentages represent the proportion of patients according to place of birth and current residence in relation to the total number of patients included in the dataset.

Although hepatitis Delta is considered a rare and neglected condition, its occurrence is almost always associated with poor clinical outcomes, including fulminant hepatic failure or rapid progression to cirrhosis and hepatocellular carcinoma^10^. In Brazil, its occurrence is largely restricted to the northern region, with approximately 73% of cases reported in the state of Amazonas^10^ ^,^ ^11^. Over a ten-year period, FMT-HVD was responsible for providing care to nearly all reported cases during this time, as shown in Figure 1. Hepatitis cases are uncommon in the southern part of Amazonas; the high number of cases observed in Manaus reflects patients seeking care at FMT and the significant migration from the interior to the capital in search of employment and better living conditions. This complex phenomenon, driven by the pursuit of improved quality of life, access to essential services, and job opportunities, was intensified during the 1960s with the establishment of the Manaus Free Trade Zone^12^, as shown in Table 1. 

**TABLE 1:** Key epidemiological indicators of hepatitis B and hepatitis D in Amazonas and Brazil.

Epidemiological indicator	Amazonas / Northern Brazil	Brazil overall
Endemicity pattern of HBV	Moderate endemicity persists despite >30 years of universal vaccination	Heterogeneous endemicity
Proportion of HBV cases reported annually	Northern region accounts for ~15-20% of cases	100%
Estimated proportion of undiagnosed HBV cases	High, particularly in riverine and Indigenous populations	~70% undiagnosed
Burden of chronic HBV and HDV carriers	High, with sustained demand for outpatient care and transplant evaluation	Significant
Distribution of hepatitis D cases	Largely restricted to the Northern region	Rare nationwide
Proportion of hepatitis D cases attributed to Amazonas	~73% of Brazilian cases	100%
Clinical severity of HDV infection	Frequent progression to fulminant hepatitis, cirrhosis, and HCC	Similar pattern, lower frequency
Geographic distribution within Amazonas	Concentrated in Manaus, reflecting migration and referral to FMT-HVD	Not applicable

### Historical background

**FIGURE 2:**
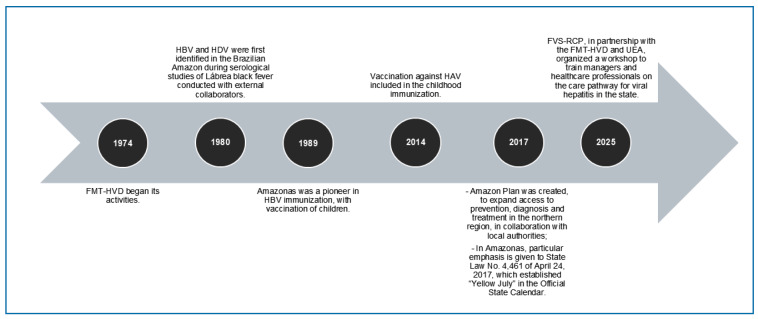
Timeline of key events in the recognition, diagnosis, and control of hepatitis B and D in the Western Amazon.

The Fundação de Medicina Tropical Dr. Heitor Vieira Dourado (FMT-HVD), originally known as the “Tropical Hospital of Manaus,” began its activities in 1975 and played a decisive role from the late 1960s through the 1970s in providing care for patients affected by a febrile ictero-hemorrhagic disease that devastated riverine populations along the Purus, Juruá, and upper Solimões Rivers. This condition, initially referred to as “Lábrea black fever,” ^13^ ^-^ ^15^ was later characterized as an HDV superinfection in individuals chronically infected with HBV, even before the availability of commercial assays for hepatitis B surface antigen (HBsAg). Notably, these investigations occurred shortly after the identification of HBV by Baruch Blumberg in Australian Aboriginal populations^16^ and the description of HDV by Mario Rizzetto^17^. Nevertheless, the circulation of HBV and HDV in the Brazilian Amazon was only definitively characterized in the 1980s, when HBsAg was detected through serological studies conducted in collaboration with national and international research groups, and HDV antigen was identified in samples from patients with Lábrea black fever^15^ ^,^ ^18^ , as shown in Figure 2. 

## RESOURCES AVAILABLE IN THE STATE OF AMAZONAS FOR THE TREATMENT AND CONTROL OF HEPATITIS B AND DELTA

Viral hepatitis represents a significant challenge for healthcare systems worldwide, with a substantial impact on morbidity and mortality^19^. The Western Amazon region, which includes the state of Amazonas, has a high number of cases of HBV/HDV co-infection , the most severe form of chronic viral hepatitis^20^. This epidemiological scenario, documented in multiple studies over the years^21^ ^,^ ^22^, requires an organized healthcare network that is capable of providing continuous services, from prevention to complex treatment.

In Brazil, the Unified Health System (SUS) is responsible for guaranteeing access to the treatment and control of viral hepatitis through programs such as the National Program for the Prevention and Control of Viral Hepatitis (PNHV)^23^. In the state of Amazonas, however, the implementation of these policies faces unique challenges, including the state’s vast territorial expanse, its low population density, and the presence of isolated communities, which directly affect the distribution of and access to healthcare resources^24^.

The healthcare structure for viral hepatitis in Amazonas is multifaceted and follows the guidelines of the PNHV. Resources are distributed across different levels of complexity, with prevention as the cornerstone of control strategies. In Amazonas, resources for this purpose include the hepatitis B vaccine, the primary prevention tool, which is universally available through the SUS. Historically, Amazonas was a pioneer in immunization, with vaccination of children beginning in the western part of the state in 1989, even before its widespread incorporation into the national vaccine schedule^23^. Vaccination against hepatitis A has also been included in the childhood immunization schedule since 2014^23^. The PNHV guides actions in the state. In addition, the “Amazon Plan” was created in 2017, an initiative by the Ministry of Health to expand access to prevention, diagnosis and treatment in the northern region, in collaboration with local authorities^25^. The Fundação de Vigilância em Saúde do Amazonas - Dra. Rosemary Costa Pinto (FVS-RCP/AM) monitors hepatitis cases, which are mandatorily reported in the Notifiable Diseases Information System (SINAN). The institution regularly publishes epidemiological bulletins to support public health actions. This monitoring is essential for guiding public policies, through the State Viral Hepatitis Program in partnership with the Municipal Health Departments, although it faces challenges such as underreporting, especially in remote areas. National campaigns are held annually, such as “Yellow July”, which was created under Federal Law No. 13,802/2019 and is aimed at raising awareness about the prevention, diagnosis and treatment of hepatitis. In Amazonas, particular emphasis is given to State Law No. 4,461 of April 24, 2017, which established “Yellow July” in the Official State Calendar.

The diagnostic network in Amazonas has a two-level structure, with broad availability for screening and centralization for specialized tests for confirmation and monitoring of treatment. Rapid testing is the most widely distributed diagnostic resource, available in all health regions of Amazonas^25^. In 2018, the state had 401 healthcare facilities^25^. Currently, the majority are in Manaus, including 204 basic health units, family health units, polyclinics and a mobile unit that perform rapid tests for hepatitis B, hepatitis C, HIV, and syphilis. Access to specialized tests, such as viral load detection (molecular biology), is centralized at designated collection points^25^. Currently, in Amazonas, these tests are processed in only three laboratories: two in the capital-at the FMT-HVD and at the Central Public Health Laboratory (LACEN-AM/FVS-RCP)-and one in Tabatinga, at the Border Laboratory of the Amazonas Secretariat for Health Surveillance (LAFRON/FVS-RCP). This centralization represents a significant barrier to continuity of care.

Treatment for chronic viral hepatitis is provided by SUS, but the availability of specialized services in Amazonas is limited and concentrated. Patient treatment and follow-up are carried out in specialized care services and hospitals. However, these services are available in only half of the state’s health regions and are heavily concentrated in the capital and its surrounding areas^25^. The state follows the National Clinical Protocols and Therapeutic Guidelines, which guarantee access to modern therapies, such as direct-acting antivirals for hepatitis C^23^. Efforts are underway to decentralize medication dispensing to primary health care facilities in order to facilitate user access^23^. In 2025, the FVS-RCP, in partnership with the FMT-HVD and the Universidade do Estado do Amazonas (UEA), organized a workshop to train managers and healthcare professionals on the care pathway for viral hepatitis in the state, aiming to improve access to treatment as well as other health measures for the prevention of vertical transmission of hepatitis B and C. 

The effectiveness of available resources is limited by challenges that are intrinsic to the Amazonian context. Geographic and logistical barriers, including the region’s vast territorial extent, long distances and reliance on river transport, hinder patient travel and the distribution of supplies, impacting timely access to diagnosis and treatment^25^ ^,^ ^26^. The centralization of care, which concentrates medium- and high-complexity services in Manaus, creates inequalities in access for populations in the interior, potentially resulting in delayed diagnosis and underreporting of cases^24^ ^,^ ^25^. In Indigenous and riverine populations, which are more vulnerable and specific, there is a high prevalence of hepatitis B and D^21^ ^,^ ^25^. The isolation of these communities represents an additional obstacle to the implementation of continuous control strategies.

Despite the obstacles and limitations, in the first half of 2025, Amazonas recorded a 34.8% reduction in viral hepatitis cases compared with the same period of the previous year^11^. The Ministry of Health bulletin^7^ also reported that deaths from hepatitis B in Amazonas decreased by 18.5% between 2014 and 2024, demonstrating that the state’s efforts at control and its interventions have yielded positive results.

In summary, the state of Amazonas has a resource structure for the control of viral hepatitis that is aligned with national policies, with a well-distributed network for prevention and initial diagnosis in the seats of its municipalities. However, the strong centralization of confirmatory diagnostic and specialized treatment services, combined with geographic and logistical barriers, compromises the comprehensiveness and equity of care. Overcoming these challenges requires the implementation of strategies to decentralize care, strengthening of primary health care and the development of care models adapted to regional realities, especially for the most vulnerable populations^23^ ^,^ ^25^. Continued investment in surveillance and evaluation of the service is essential to support decision-making, enhance the health system’s response to this significant public health burden in the region, and will ensure that the WHO targets for the diagnosis and control of viral hepatitis are met by 2030.

Overall, the current health-care network for viral hepatitis in Amazonas presents important strengths and persistent structural weaknesses. Key strengths include the alignment with national policies, the long-standing implementation of universal hepatitis B vaccination, the broad availability of rapid screening tests across health regions, and the existence of reference centers with expertise in HBV/HDV management. Conversely, major weaknesses remain, particularly the strong centralization of confirmatory diagnostics and specialized care in the capital, limited availability of treatment services in the interior, geographic and logistical barriers to access, and ongoing challenges related to underdiagnosis and underreporting in riverine and Indigenous populations. Together, these factors highlight both the advances achieved and the critical gaps that must be addressed to ensure equitable and timely care throughout the state.

## EVOLUTION OF KNOWLEDGE FOR THE TREATMENT AND CONTROL OF HEPATITIS B AND DELTA

This history of lacking modern diagnostic resources and dependence on partner institutions reflects the legacy of unjust scientific colonialism, often imposed by the health authorities themselves. Recently, the Brazilian Ministry of Health issued a decree stipulating that all HDV viral load tests must be performed at the FIOCRUZ unit in Rondônia, using an in-house molecular technique^27^, with the originating laboratories responsible for the logistics and costs of the operation. This occurs even though a validated commercial test exists, which could be performed on equipment already used for HIV viral load testing, including in border laboratories.

From a constructive perspective, addressing these limitations will require the validation and incorporation of commercially available HDV diagnostic assays in regional laboratories, enabling their use on existing molecular platforms already employed for HIV and other infections. In parallel, the decentralization of diagnostic technologies and strategic distribution of point-of-care or rapid testing tools, particularly in rural and remote settings, could reduce dependence on centralized facilities and improve timely access to care. Investments in local infrastructure and workforce training are essential to support these approaches and to generate context-specific data capable of informing surveillance, clinical management, and public health decision-making in the Amazonian region.

Advances in the diagnosis of HBV infection over the slightly more than fifty years since its discovery range from point-of-care tests to sophisticated molecular assays. These advances have been instrumental in the characterization and quantification of all viral molecules, as well as in elucidating the mechanisms of viral replication and interaction with the human host. However, it will likely take at least another decade before these advances significantly benefit patients in the western Amazon.

Unlike hepatitis C virus (HCV), for which a molecule capable of eradicating the virus from hepatocytes in over 90% of cases was developed within a decade^28^, HBV treatment has largely relied on drugs designed for HIV, due to similarities in the viral molecules that are involved in replication. However, more recently, new medications targeting specific steps of HBV replication are being designed and tested in various phases of clinical trials; although it will still be a few years before the results of these studies are available. Treatment options are expanding to target both the host immune response and the viral molecules involved in cell entry, as well as particles produced during viral replication and assembly. Antiviral treatment for HDV still depends on the development of new strategies to address HBV, although some results using HBV entry inhibitors, nucleoside or nucleotide analogues, and pegylated interferon have shown promising outcomes^29^ ^,^ ^30^. 

Considering that HBV emerged more than 80 million years ago, it may be overly optimistic to think that we could unravel its mysteries in just 50 years. Nevertheless, we believe that new approaches can be anticipated, and that some previously unknown aspects of virus-host interactions may be revealed as scientific and technological knowledge advances.

Major advances in the control of hepatitis B and D have been linked to the implementation of universal vaccination, which uses a highly effective, stable, low-cost vaccine with a logistics system adaptable to diverse geographic contexts. This has been exemplified in the western Amazon among isolated Indigenous populations, riverine communities and vulnerable migrant populations living on the outskirts of major cities in northern Brazil. Although various programs have been successful in controlling the transmission chain^6^, the prevalence of infection often shows increasing trends as new cases are identified through rapid testing campaigns. This highlights the need to decentralize care for chronic carriers, modernize outpatient clinics, train new professionals, implement multidisciplinary activities and explore new therapeutic options.

## BARRIERS AND FACILITATORS FOR THE TREATMENT AND CONTROL OF HEPATITIS B AND DELTA IN AMAZONAS

In Brazil, since the creation of the PNHV within the SUS in 2002, strategies for addressing viral hepatitis have focused on prevention and, more recently, on guidelines for organizing the healthcare network^23^. 

### Barriers

However, the distribution of health services for hepatitis care remains uneven, resulting in persistent challenges in translating technological advances in diagnosis and treatment into equitable access. These disparities are shaped by socioeconomic heterogeneity, the organization of the healthcare network and weaknesses in policy directives that are aimed at expanding universal access and ensuring equity and comprehensiveness in public healthcare. 

Other factors include the genetic variability of HBV, individual responses to treatment, the persistence of transmission, limited access to testing, and social stigma, all of which can hinder early diagnosis and appropriate care. It is also important to note that in the state of Amazonas, these challenges are further amplified by the vast territory and hard-to-reach areas, which complicate the provision of health services, including testing, treatment and follow-up for people with hepatitis B. Regarding hepatitis D, the absence of a specific vaccine makes its eradication an even more complex challenge.

### Facilitators

The FMT-HVD serves as a reference center for the diagnosis and treatment of viral hepatitis, providing care through a multidisciplinary team. In addition, it conducts scientific research that can deepen the understanding of patient profiles and the most effective treatments. It is important to highlight the foundation’s crucial role in addressing hepatitis B, as this ensures patients receive the most effective management, preventing complications such as cirrhosis and liver cancer.

## RESEARCH PRIORITY AGENDA FOR 2026-2036

Since its inception, the FMT-HVD has been involved in the clinical and epidemiological investigation of viral hepatitis in Amazonas, including the etiological identification of Lábrea black fever and the role of HBV/HDV superinfection. Below, we outline priority research gaps to be addressed in the context of the Amazon over the next decade.

### In epidemiology and surveillance

- Generate valid and timely estimates of the occurrence and burden of HBV, HCV and HBV/HDV (prevalence, incidence, mortality, DALYs) by municipality and population group (riverine communities, rural populations, urban periphery residents, Indigenous peoples, incarcerated individuals, people living with HIV, children, and pregnant women).
- Assess the genotypes/lineages of HBV, HCV and HDV circulating in Amazonas and their association with clinical outcomes and treatment response.
- Study the phylodynamics of HBV, HCV and HDV to strengthen molecular surveillance integrated into the public health system and guide prevention, screening and control strategies through the integration of clinical, laboratory, epidemiological and genomic data. In this context, FMT-HVD has been actively engaged in ongoing molecular studies on hepatitis B and D, supported by established research partnerships with other institutions across Brazil.

### In clinical and translational research

- Generate causal evidence on the biological, social, cultural, environmental and occupational determinants of HBV, HCV and HBV/HDV, and identify high-risk clusters (hotspots) to support prevention, early detection, linkage to care and the reduction of health inequities. In parallel, FMT-HVD is conducting ongoing studies based on the systematic collection and analysis of epidemiological data from individuals receiving care at the institution, contributing to a more detailed characterization of affected populations.
- Conduct clinical trials with new pharmacological therapies to generate robust evidence on their efficacy and safety, taking into account the clinical, biological and contextual specificities of the region. In this regard, FMT-HVD conducts clinical trials for the treatment of viral hepatitis in partnership with pharmaceutical industries, contributing to the evaluation of innovative therapeutic strategies in real-world endemic settings.
- Study clinical and laboratory markers that predict progression to fibrosis, cirrhosis and hepatocellular carcinoma in patients with HBV and in patients with HBV/HDV. Ongoing human genetics studies addressing these outcomes are currently being conducted at FMT-HVD in collaboration with other national and international research institutions, strengthening multicenter approaches to disease progression and risk stratification

### In social and cultural determinants

- Incorporate variables related to climate change, deforestation and unplanned urbanization to understand how these factors may influence the dynamics of hepatitis in the Amazon.
- Assess the logistical and sociocultural barriers to HBV vaccination that may compromise coverage and the timely administration of the birth dose and the complete vaccination schedule.
- Studies evaluating testing workflows in health units by municipality.
- Studies evaluating the care cascade in municipalities: screening → confirmation → staging → treatment initiation → virologic cure (HCV)/suppression (HBV) → follow-up.
- Identify barriers to accessing health services by territory (urban-peripheral, riverine, rural, Indigenous).
- Cost studies on viral hepatitis in Amazonas from the perspective of patients and their families, as well as from the health service perspective.
- Research into the cost-effectiveness of strategies such as therapeutic decentralization (prescription/dispensing at the primary healthcare level) and teleconsultation with hepatology and infectious disease specialists.
- Studies using digital tools to assess treatment adherence.
- Study the stigma associated with viral hepatitis and its impact on testing and treatment adherence.
- Studies evaluating culturally sensitive health communication.
- Evaluate the therapeutic itinerary of patients in the Amazon region.
- Map the traditional practices and knowledge of riverine communities, rural populations, residents of the urban periphery and Indigenous peoples in regard to HBV and HCV.
- Map social participation, local governance and co-management of care in each municipality.

The FMT-HVD has maintained its role in leading research on viral hepatitis in the state of Amazonas, thereby contributing evidence to guide the management of these infections. In recent years, it has also served as a clinical trial center for studies on new drugs for hepatitis B and D, focusing on the Amazonian population, which remains underrepresented in clinical trials. Continued and targeted investment in viral hepatitis research in the context of the Amazon is essential. These resources should support clinical-epidemiological studies aimed at understanding the distribution, determinants and impact of these infections; policy and management research to guide effective prevention, diagnosis and treatment strategies, and social and cultural studies to deepen the analysis of structural determinants and promote inclusive and equitable actions. This integrated approach is essential in order to reduce inequalities, improve public policies and strengthen the regional response to this significant health challenge.

## CONCLUSIONS

The history of the Fundação de Medicina Tropical Dr. Heitor Vieira Dourado (FMT-HVD) is closely intertwined with the recognition and characterization of hepatitis B and hepatitis D in the Brazilian Amazon, playing a central role in the diagnosis, clinical follow-up, and care of individuals living with these infections. Despite its longstanding contribution and the existence of a specialized outpatient clinic dedicated to viral hepatitis, significant challenges persist, particularly regarding resource limitations, geographic barriers, and the concentration of specialized services in the capital.

Progress toward the elimination of viral hepatitis by 2030 has been gradual in the Amazonian context. Nevertheless, advances in therapeutic strategies for HBV and HDV, along with improvements in surveillance, early diagnosis, and health-care organization, offer promising opportunities to strengthen disease control. Sustained investment in research, health-care infrastructure, and context-adapted care models will be essential to address regional inequities and to translate scientific advances into tangible public health gains for populations disproportionately affected by viral hepatitis in the Amazon.

## Data Availability

Research data is available in the body of the article.

## References

[1] Braga WSM, Oliveira CMC de, Araújo JR de, Castilho M da C, Rocha JM, Gimaque JB de L (2014). Chronic HDV/HBV co-infection: Predictors of disease stage - a
case series of HDV-3 patients. J Hepatol.

[2] Cabezas C, Trujillo O, Balbuena J, Peceros FM, Terrazas M, Suárez M (2020). Decrease in the prevalence of hepatitis B and D virus infections
in an endemic area in Peru 23 years after the introduction of the first
pilot vaccination program against hepatitis B. PLoS ONE.

[3] Duarte MC, Cardona NE, Poblete F, González K, García M, Pacheco M (2010). A comparative epidemiological study of hepatitis B and hepatitis
D virus infections in Yanomami and Piaroa Amerindians of Amazonas State,
Venezuela. Trop Med Int Health.

[4] Montoya-Guzman M, Martinez J, Castro-Arroyave D, Rojas C, Navas MC (2023). Epidemiology and Genetic Diversity of Hepatitis B Virus and
Hepatitis Delta Virus Infection in Indigenous Communities in
Colombia. Microorganisms.

[5] Manock SR, Kelley PH, Hyams KC, Douce RW, Smalligan RD, Watts DM (2000). An outbreak of fulminant hepatitis delta in the Waorani, an
indigenous people of the Amazon basin of Ecuador. Am J Trop Med Hyg.

[6] Cabezas C, Braga W (2020). Hepatitis B Virus and Delta Infection: Special Considerations in
the Indigenous and Isolated Riverside Populations in the Amazon
Region. Clinical Liver Disease.

[7] Ministério da Saúde (BR). Secretaria de Vigilância em Saúde (2024). Boletim Epidemiológico: Hepatites Virais.

[8] Razavi-Shearer D, Gamkrelidze Ivane, Pan CQ, Jia J, Berg T, Gray RT (2023). Global prevalence, cascade of care, and prophylaxis coverage of
hepatitis B in 2022: a modelling study. Lancet Gastroenterol Hepatol.

[9] World Health Organization (2022). Global health sector strategies on, respectively, HIV, viral hepatitis
and sexually transmitted infections for the period 2022-2030.

[10] Rizzetto M (2022). Hepatitis D (DELTA). New Microbiol.

[11] Fundação de Vigilância em Saúde do Amazonas (2026). Portal FVS-RCP/AM: Sala de Situação.

[12] Fonseca VN, Martins C (2023). Dialógos entre história e educação: Um olhar sobre o caso da zona
franca de manaus. EDUCAmazônia.

[13] Bensabath G, Dias LB (1983). Labrea hepatitis (Labrea black fever) and other fulminant forms
of hepatitis in Sena Madureira, Acre and Boca do Acre, Amazonas,
Brazil. Rev Inst Med Trop Sao Paulo.

[14] Casey JL, Niro GA, Engle RE, Vega A, Gomez H, McCarthy M (1996). Hepatitis B Virus (HBV)/Hepatitis D Virus (HDV) Coinfection in
Outbreaks of Acute Hepatitis in the Peruvian Amazon Basin: The Roles of HDV
Genotype III and HBV Genotype F. J Infect Dis.

[15] Fonseca JCF da, Ferreira LCL, Guerra ALP da S, Passos LM, Simonetti JP (1983). Hepatite fulminante e febre negra de Lábrea: estudo de 5 casos
procedentes de Codajás, Amazonas, Brasil. Rev Soc Bras Med Trop.

[16] Blumberg BS (1965). A “New” Antigen in Leukemia Sera. JAMA.

[17] Rizzetto M, Canese MG, Aricò S, Crivelli O, Trepo C, Bonino F (1977). Immunofluorescence detection of new antigen-antibody system
(delta/anti-delta) associated to hepatitis B virus in liver and in serum of
HBsAg carriers. Gut.

[18] Andrade ZA, Santos JB, Prata A, Dourado H (1983). Histopatologia da hepatite de Lábrea. Rev Soc Bras Med Trop.

[19] Stanaway JD, Flaxman AD, Naghavi M, Fitzmaurice C, Vos T, Abubakar I (2016). The global burden of viral hepatitis from 1990 to 2013: findings
from the Global Burden of Disease Study 2013. Lancet.

[20] Costa PL de S, Andrade MAH de, Silva VV, Costa ACC, Silva AMF da, Oliveira P da S (2020). Coinfecção da Hepatite B e Delta na Amazônia: Artigo de
atualização. Revista Eletrônica Acervo Saúde.

[21] Braga WSM, Brasil LM, Souza RAB de, Castilho M da C, Fonseca JC da (2001). Ocorrência da infecção pelo vírus da hepatite B (VHB) e delta
(VHD) em sete grupos indígenas do Estado do Amazonas. Rev Soc Bras Med Trop.

[22] Braga WSM, Silva EB da, Souza RAB de, Tosta CE (2005). Soroprevalência da infecção pelo vírus da hepatite B e pelo
plasmódio em Lábrea, Amazonas: estimativa da ocorrência de prováveis
coinfecções. Rev Soc Bras Med Trop.

[23] Gleriano JS, Chaves LDP, Pantoja VJ da C, Caminada S (2023). 20 anos do programa nacional para a prevenção e o controle das
hepatites virais: processo histórico e contribuições para a
gestão. Administração Pública e Gestão Social.

[24] Garnelo L (2019). Especificidades e desafios das políticas públicas de saúde na
Amazônia. Cad Saude Publica.

[25] de Almeida EC, Gleriano JS, Pinto FKA, Coelho RA, Vivaldini SM, Gomes JNN (2019). Acesso à atenção às hepatites virais: distribuição de serviços na
região Norte do Brasil. Rev Bras Epidemiol.

[26] Garnelo L, Sousa ABL, Silva COD (2017). Health regionalization in Amazonas: progress and
challenges. Cien Saude Colet.

[27] (2024). Departamento de HIV, Aids, Tuberculose, Hepatites Virais e Infecções
Sexualmente Transmissíveis.

[28] Baumert TF, Berg T, Lim JK, Nelson DR (2019). Status of Direct-Acting Antiviral Therapy for Hepatitis C Virus
Infection and Remaining Challenges. Gastroenterology.

[29] Wedemeyer H, Aleman S, Brunetto MR, Blank A, Andreone P, Bogomolov P (2023). A Phase 3, Randomized Trial of Bulevirtide in Chronic Hepatitis
D. N Engl J Med.

[30] Borzacov LMP, de Figueiredo Nicolete LD, Souza LFB, Dos Santos AO, Vieira DS, Salcedo JMV (2016). Treatment of hepatitis delta virus genotype 3 infection with
peg-interferon and entecavir. Int J Infect Dis.

